# A Genome-Wide Analysis of the Jasmonic Acid Biosynthesis Gene Families in Peanut Reveals Their Crucial Roles in Growth and Abiotic Stresses

**DOI:** 10.3390/ijms25137054

**Published:** 2024-06-27

**Authors:** Xinlei Ma, Xin Ai, Chenghua Li, Shiyu Wang, Nan Zhang, Jingyao Ren, Jing Wang, Chao Zhong, Xinhua Zhao, He Zhang, Haiqiu Yu

**Affiliations:** Peanut Research Institute, College of Agronomy, Shenyang Agricultural University, Shenyang 110161, China

**Keywords:** abiotic stress, genome-wide, jasmonic acid biosynthesis, peanut

## Abstract

Abiotic stress is a limiting factor in peanut production. Peanut is an important oil crop and cash crop in China. Peanut yield is vulnerable to abiotic stress due to its seeds grown underground. Jasmonic acid (JA) is essential for plant growth and defense against adversity stresses. However, the regulation and mechanism of the jasmonic acid biosynthesis pathway on peanut defense against abiotic stresses are still limitedly understood. In this study, a total of 64 genes encoding key enzymes of JA biosynthesis were identified and classified into lipoxygenases (AhLOXs), alleno oxide synthases (AhAOSs), allene oxide cyclases (AhAOCs), and 12-oxo-phytodienoic acid reductases (AhOPRs) according to gene structure, conserved motif, and phylogenetic feature. A *cis*-regulatory element analysis indicated that some of the genes contained stress responsive and hormone responsive elements. In addition to proteins involved in JA biosynthesis and signaling, they also interacted with proteins involved in lipid biosynthesis and stress response. Sixteen putative Ah-miRNAs were identified from four families targeting 35 key genes of JA biosynthesis. A tissue expression pattern analysis revealed that AhLOX2 was the highest expressed in leaf tissues, and AhLOX32 was the highest expressed in shoot, root, and nodule tissues. AhLOX16, AhOPR1, and AhOPR3 were up-regulated under drought stress. AhLOX16, AhAOS3, AhOPR1, and AhAOC4 had elevated transcript levels in response to cold stress. AhLOX5, AhLOX16, AhAOC3, AhOPR1, and AhOPR3 were up-regulated for expression under salt stress. Our study could provide a reference for the study of the abiotic stress resistance mechanism in peanut.

## 1. Introduction

Plant hormones play important roles in the regulation of plant responses to various stimuli and stresses [1]. Jasmonic acid (JA) is an oxidized fatty acid derivative, which together with its derivatives and active precursor 12-oxo-phytodienoic acid (12-OPDA) are collectively known as jasmonates (JAs) [2]. Jasmonic acid can inhibit root growth, accelerate fruit ripening, and regulate flowering plant development [3,4]. As a signal transduction molecule, it is closely related to plant resistance to biological (injury, insect bite, etc.) and abiotic stresses (drought, low temperature, salinity, and wounding), and may participate in the crosstalk between various hormones [5,6]. The biosynthesis of JA is performed in the cytoplasm, chloroplast, and peroxisome [4]. Firstly, α-linolenic acid (α-LeA) is catalyzed in the chloroplast and transported to peroxisomes as a synthetic feedstock. Subsequently, cis-(+)-12-oxo-phytodienoic acid (12-OPDA) is generated via lipoxygenase (LOX; EC1.13.11.12), alleno oxide synthase (AOS; EC4.2.1.92) and allene oxide cyclase (AOC; EC5.3.99.6). Then, with the help of 12-oxo-phytodienoic acid reductase (OPDA reductase, OPR; EC1.3.1.42), 12-OPDA is reduced to OPC-8:0, followed by three rounds of β-oxidation [3,7]. The final product of β-oxidation is (3R,7S)-JA (OPC-2:0), which can form the more stable isomer (3R,7R)-JA [8,9]. These two isomers of JA coexist in plants. Eventually, JA diffuses into the cytoplasm, and various derivatives are formed through different modifications [10,11,12].

Lipoxygenase (LOX; EC1.13.11.12), a non-heme iron-containing oxygenase, can catalyze the oxygenation of polyunsaturated fatty acids to produce hydroperoxides [13]. The lipoxygenase domain and PLAT_LH2 domain are the core components of LOXs. According to the binding site of its substrate during oxygenation, LOXs in plants can be divided into 9-LOXs and 13-LOXs, of which 13-LOXs can be further divided into type I and type II 13-LOXs [14]. Alleno oxide synthase (AOS; EC4.2.1.92) belongs to the CYP74 subfamily within the cytochrome P450 superfamily. Alleno oxide synthases have been verified to play a crucial role in the biosynthesis of oxylipins, bioactive compounds involved in signal and defense reactions in higher plants, mammals, and algae [15]. Alleno oxide synthases do not require molecular oxygen, nor do they require cytochrome P450 reductases that rely on NAD(P)H as co-factors, but instead use their hydrogen peroxide substrates as a source and oxygen donor for reducing equivalents [16,17]. Allene oxide cyclase (AOC; EC5.3.99.6) is a dimeric enzyme located in chloroplasts [18]. Since first cloned in tomato by Ziegler [19], AOC genes have been isolated and identified in many plant species such as *Arabidopsis*, rice, wheat, soybean, and cannabis [19,20,21,22,23,24]. 12-Oxo-phytodienoic acid reductase (OPDA reductase, OPR; EC1.3.1.42) belongs to the old yellow enzyme (OYE) family and is classified as FMN-dependent oxidant oxidase [25,26]. In 1997, OPRs were first identified in the *Arabidopsis* genome and divided into OPRI and OPRII [27]. In rice, the classification of OPRs into five classes was based on the three-dimensional structural differences and deep replication nodes of the proteins, which determine the substrate specificity and catalytic activity of the different isomeric OPR proteins [28].

It has been shown that key enzymes catalyzing JA biosynthesis play an important role in plant adaptation to adversity stress. In mung bean (*Vigna radiata*), LOXs’ activity was significantly elevated after an external application of 20% PEG-6000 [29]. Both 9-LOXs and 13-LOXs have been reported to be closely associated with plant responses to external stimuli. The 13-LOX subfamily plays a role in wounding and other defense responses [30]. At the 190 s time point, the combined lox3B-lox4A mutation reduced JA concentration damaged leaf 8 by approximately 20% relative to the wild type in *Arabidopsis*. The over-expression of LOX10 enhances tolerance to salinity stress in rice seedlings [31]. The 9-LOX subfamily, on the other hand, has been linked to pathogen defense [32]. An OPDA-like 9-LOX-derived compound, 10-OPEA (10-oxo-11-phytoenoic acid), plays an important role in maize fungal defense [33]. Alleno oxide synthases have been reported to limit JA levels in injured plants and to affect reproductive fertility. A functional mutation in AOSs results in injured Arabidopsis that shows no increase in endogenous JA levels. The wild type shows a 100-fold increase in endogenous JA levels in 1 h. Allene oxide cyclase is an important member in the biosynthesis of JA, and transgenic tobacco plants overexpressing GmAOC1 have leaves that remain green and possess higher fresh weights and normal root growth after treatment with 150 mM NaCl, whereas the wild type turns markedly yellow and has severely retarded root growth, suggesting that the over-expression of GmAOC1 improves the plant’s tolerance to salt stress [22].

Peanut (*Arachis hypogaea* L.) is an important oil crop with high nutritional and commercial value in China [34]. China is one of the world’s largest peanut producers, with more than 4.6 million hm^2^ sown area and more than 17 million tons of pod production, accounting for 40% of the global total [35]. China has gradually formed the oil crop planting structure with peanut and rape as the main elements in the development of agricultural production and increased agricultural supply has become increasingly prominent. During the growth period, peanuts suffer from a variety of natural disasters and weather, which have a greater impact on the growth and development of peanuts, often causing a decline in photosynthesis, slow growth, and serious production reduction or even extinction [36]. Under abiotic stress, photosynthesis, nutrient absorption, metabolism, growth, and final crop yield of peanut will all undergo adverse effects [37]. Therefore, clarifying the molecular mechanism of abiotic adaptation in peanut and genetic improvement for stress tolerance was crucial to safeguard national edible oil and food security. However, there are fewer reports on the regulation and mechanism of action of the JA biosynthetic pathway on peanut stress tolerance. In the present study, we identified key enzyme proteins of JA biosynthesis in allotetraploid peanut and analyzed their phylogenetic relationship, gene structure, conserved domains and motifs, chromosomal distribution, phylogenetic features, *cis*-regulatory elements, and expression profiles in response to drought, cold, and salt treatments. Our results provide a bioinformatics analysis and candidate genes that may be potentially utilized in improving crop resistance to abiotic stress.

## 2. Results

### 2.1. Identification and Characterization of JA Biosynthesis Gene Families in Arachis

**Figure 1:**
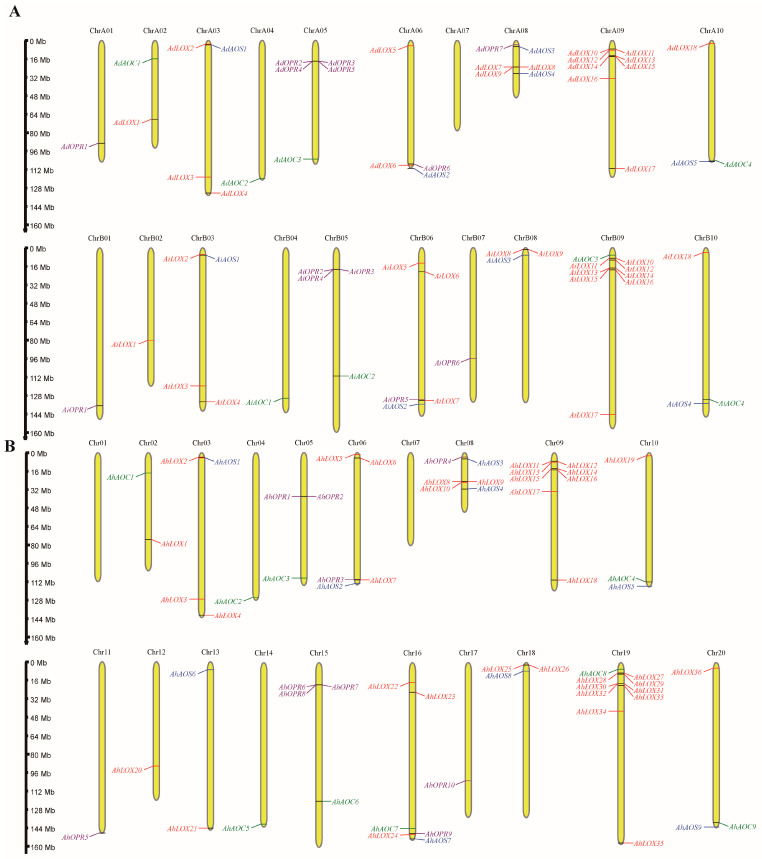
The distribution of the *LOX*, *AOS*, *AOC*, and *OPR* genes in *A. duranensis*, *A. ipaensis* (**A**), and *A. hypogaea* (**B**).

A total of 130 genes encoding key enzyme proteins of JA biosynthesis were identified in *A. duranensis*, *A. ipaensis,* and *A. hypogaea* genomes by HMMER [38] (Tables S1–S4). These genes corresponded to four gene families (lipoxygenase, LOX; allene oxide synthase, AOS; allene oxide cyclase, AOC; and 12-oxophytodienoate reductase, OPR). The remaining sequences were checked for the existence of complete domains utilizing Batch Web CD-Search Tool, Pfam, and SMART websites. A total of 72 LOXs, 18 AOSs, 17 AOCs, and 23 OPRs were finally confirmed as key enzyme proteins of JA biosynthesis and renamed based on their chromosomal locations (Figure 1). In this study, *LOX*, *AOS*, *AOC,* and *OPR* genes were unevenly distributed in nineteen of twenty chromosomes of *A. duranensis* (A genome) and *A. ipaensis* (B genome). Chromosome 19 contained the most genes and no JA biosynthesis gene was discovered in chromosome A07, 01, and 17. Many homologous genes in allotetraploid peanut were mapped to key enzyme genes of JA biosynthesis in A and B genomes. Multiple sequence alignment results demonstrated that the homologous gene pairs did have high sequence similarity (Table S5).

### 2.2. Physicochemical Properties of JA Biosynthesis Genes

In order to explore the sequence characteristics of 72 LOXs, 18 AOSs, 17 AOCs, and 23 OPRs in *Arachis*, the opening reading frame (ORF) lengths, theoretical isoelectric point (pI), and other basic information were analyzed. The length of LOX proteins varied from 509 to 477 amino acids (AAs). The molecular weights (MWs) ranged from 58.35 kDa to 53.18 kDa. The predicted isoelectric point value of LOX proteins was between 4.98 and 9.15. The instability index of LOX proteins was between 33.31 and 52.05, and the aliphatic index was between 78.96 and 93.88. All predicted LOX proteins showed a grand average of hydropathicity (GRAVY) value below zero, meaning hydrophilic protein (Table S1). The peanut AOS proteins have a maximum length of 590 AAs and a minimum of 336 AAs, with pI ranging from 5.79 to 8.91. The MW ranged from 42.8 kDa to 66.5 kDa. The instability index of AOS proteins was between 33.39 and 49.44, and the aliphatic index was between 79.29 and 94.64. All predicted AOS proteins’ GRAVY value below zero means that they were hydrophilic protein (Table S2). The AOC protein lengths ranged from 178 to 303 amino acids. The MW of AOC proteins varied from 19.78 kDa to 33.64 kDa, and the pI values ranged from 6.90 to 9.83. The instability index of AOC proteins was between 27.78 and 55.52, and the aliphatic index was between 74.78 and 98.74. All predicted AOC proteins showed a GRAVY value below zero (Table S3). The protein lengths of 23 OPR genes ranged from 339 AAs to 573 AAs. The MW ranged from 37.56 kDa to 64.44 kDa and pI of these proteins ranged from 5.52 to 6.84. The instability index of OPR proteins was between 29.86 and 43.17, and the aliphatic index was between 74.01 and 84.06. All predicted OPR proteins’ GRAVY value below zero means that they were hydrophilic protein (Table S4). Subcellular localization results demonstrated that all LOX protein was located in the cytoplasm, 12 AOSs worked in the endoplasmic reticulum, and 5 AOS and 17 AOC proteins were located in the chloroplast. All OPR proteins were located in the cytoplasm apart from AdOPR1, AiOPR1, and AhOPR5 (Tables S1–S4).

### 2.3. Phylogenetic Analysis of JA Biosynthesis Gene Families

**Figure 2:**
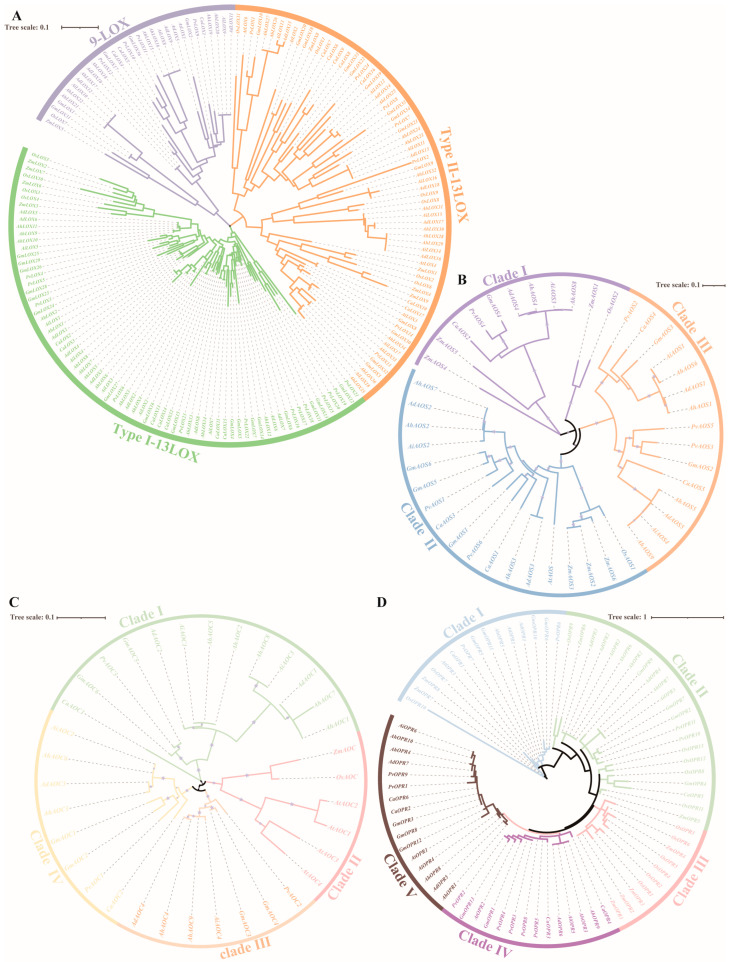
The phylogenetic analysis of proteins implicated in JA biosynthesis-related enzymes in *Arabidopsis thaliana*, *Oryza sativa*, *Zea mays*, *Glycine max*, *Phaseolus vulgaris*, Cicer arietinum, *A. duranensis*, *A. ipaensis*, and *A. hypogaea*. A phylogenetic tree was created using the maximum-likelihood (ML) method with 1000 replications by MEGA11 software (version v11.0.13). (**A**) AhLOXs; (**B**) AhAOSs; (**C**) AhAOCs; (**D**) AhOPRs.

To analyze the classification and evolutionary relationships among the protein members of JA biosynthesis gene families in different species, four phylogenetic trees were constructed utilizing the HMM search tool based on the protein sequences in *Arabidopsis thaliana*, *Oryza sativa*, *Zea mays*, *Glycine max*, *Phaseolus vulgaris*, *Cicer arietinum*, *A. duranensis*, *A. ipaensis*, and *A. hypogaea* (Figure 2). A multiple-sequence alignment was established among 18 AdLOXs, 18 AiLOXs, 36 AhLOXs, 6 AtLOXs, 11 OsLOXs, 9ZmLOXs, 36 GmLOXs, 24 PvLOXs, and 17 CaLOXs using clustalW software (Version 2.1). 

According to previous studies in Arabidopsis, LOX proteins could be classified into three distinct subfamilies: 9-LOX, type I 13-LOX, and type II 13-LOX (Figure 2A). AhLOX had 8, 14, and 14 members in three subfamilies, respectively. In the 9-LOX subfamily, AdLOX9-12, AiLOX8-10, and AhLOX15-22 were closely related to GmLOX1-2, GmLOX17, GmLOX31, GmLOX36, PvLOX9-12, CaLOX1, and CaLOX4-5. AdLOX1-8, AiLOX1-7, and AhLOX1-14 had the highest relationship with GmLOX4-7, GmLOX11-16, GmLOX23-29, PvLOX3-6, PvLOX15-23, CaLOX2, and CaLOX11-15 in type-I 13-LOX. In the same trend as in the type I 13-LOX family, AdLOX, AiLOX, and AhLOX were closely related to GmLOXs, PvLOXs, and CaLOXs, and the largest number of AtLOXs, OsLOXs, and ZmLOXs were gathered in the type II 13-LOX family. In general, peanut LOX is closely related to soybean, chickpeas, and common beans, which belong to *Fabaceae*.

The nine AhAOS members were non-randomly distributed in clade I, II, and III (Figure 2B). The phylogenetic relationship analysis revealed that AdAOS4, AiAOS3, AhAOS4, and AhAOS8 were tightly related to OsAOS2, ZmAOS1, ZmAOS4-5, GmAOS4, PvAOS4, and CaAOS2. There were three ZmAOSs in clade I and II, respectively. There were the largest number of AhAOSs in clade III. It is noteworthy that the only AtAOS appeared in clade II and AtAOSs, OsAOSs, and ZmAOSs were not found in clade III. 

A total of 34 AhAOC proteins were identified and classified into four clades: clade I, II, III, and IV (Figure 2C). AdAOC1-2, AiAOC1, AiAOC3, AhAOC1-2, AhAOC5, and AhAOC7-8 were in close proximity to GmAOC5-6, PvAOC3, and CaAOC1. Four AtAOCs, one OsAOC, and one ZmAOC were all clustered, and no members of other species were discovered in clade II. Clade III and IV contained the same amount of *Arachis* AOC members. Only AOCs of *Fabaceae* existed in clade I, III, and IV; this phenomenon suggested that *Arachis* AOC members had high homology with AOC in *Glycine max*, *Phaseolus vulgaris,* and *Cicer arietinum*. 

According to the branches of the phylogenetic tree and classification of OPR gene family members in *Arabidopsis thaliana* and *Oryza sativa* [39,40], five, four, nine, one, two, six, six, six, and five members of the OPR family were identified and divided into five clades in *A. duranensis*, *A. ipaensis*, *A. hypogaea*, *Arabidopsis thaliana*, *Oryza sativa*, *Zea mays*, *Glycine max*, *Phaseolus vulgaris,* and *Cicer arietinum*, respectively (Figure 2D). All species in clade I had at least one OPR member. Only OsOPRs and ZmOPRs remained in clade III. Three AtOPR proteins were distributed in clade I, IV, and V. Four AhOPRs existed in clade V. Taken together, compared to *Arabidopsis thaliana*, *Oryza sativa*, and *Zea mays*, the results indicated that peanut key enzyme proteins of JA biosynthesis had higher homology with a family member in *Glycine max*, *Phaseolus vulgaris*, and *Cicer arietinum*.

### 2.4. Domain Characterization, Gene Structure, Conserved Motif Distribution, and Protein Structure of JA Biosynthesis Gene Families

**Figure 3:**
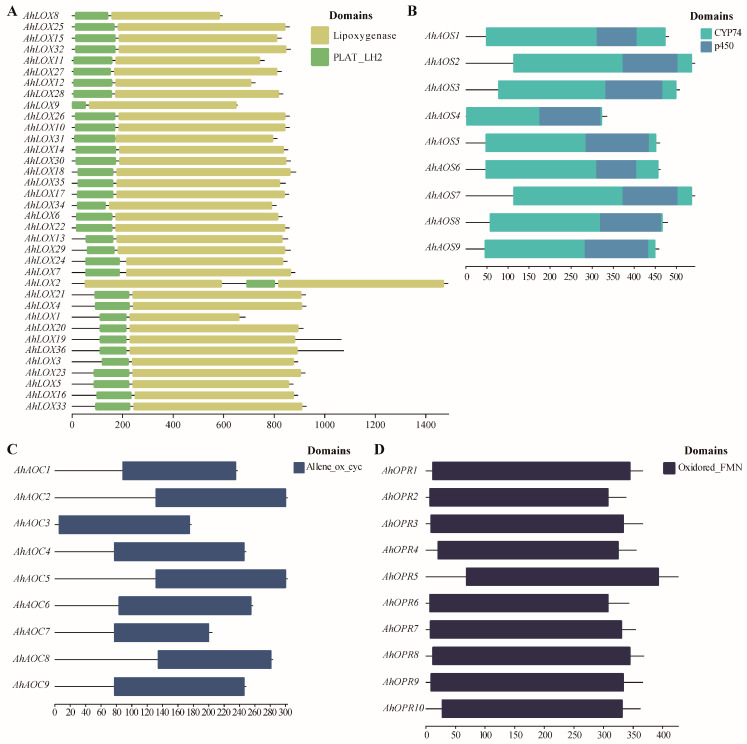
Distribution of conserved domains of JA biosynthesis-related enzyme genes in *A. hypogaea*. (**A**) AhLOXs; (**B**) AhAOSs; (**C**) AhAOCs; (**D**) AhOPRs.

In order to determine the presence of conserved domains, key enzyme genes of JA biosynthesis were filtrated by the CDD database (Figure 3). The results confirmed that the lipoxygenase domain, p450 domain, Allene_ox_cyc domain, and Oxidored_FMN domain were successfully detected in AhLOXs, AhAOSs, AhAOCs, and AhOPRs, respectively. As one of the branches of the p450 superfamily, the CYP74 domain is the typical characteristic and distinguishing basis of the AOS gene family. At the same time, a lipoxygenase domain and a PLAT_LH2 domain exist simultaneously to be considered a member of the LOX family. The analysis revealed that all members of AhLOXs, AhAOSs, AhAOCs, and AhOPRs contained characteristic domains of their family.

**Figure 4:**
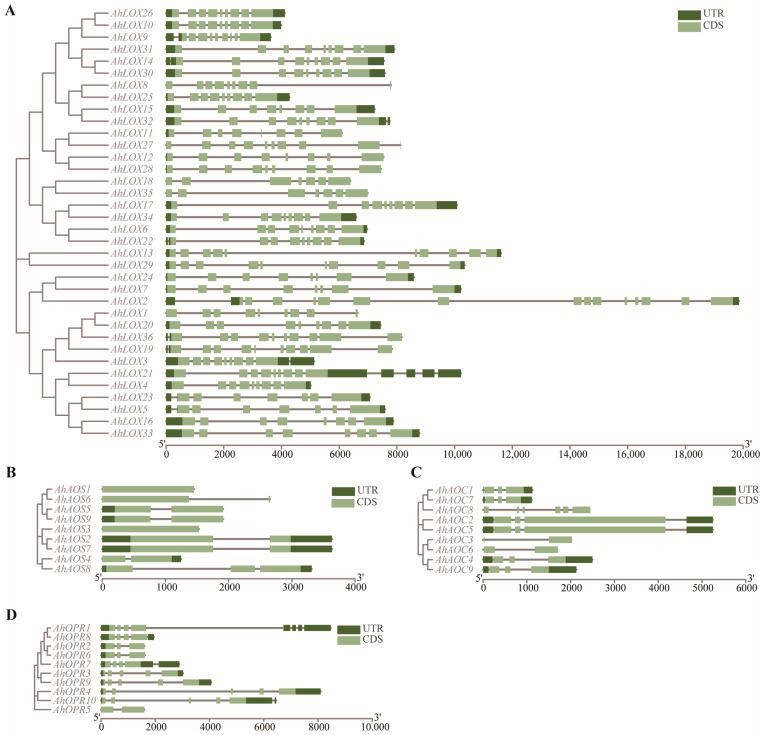
Distribution and composition of exons–introns of JA biosynthesis-related enzyme genes in *A. hypogaea*. (**A**) AhLOXs; (**B**) AhAOSs; (**C**) AhAOCs; (**D**) AhOPRs.

The number of introns and the way introns are inserted may be the cause of gene evolution and functional differentiation. In this study, the exon–intron organizations of all the identified JA biosynthesis genes were examined to gain more insight into the evolution in allotetraploid peanut. The phylogenetic tree and gene structure were integrated together as shown in Figure 4. The number of introns of AhLOX varied from 6 to 14 (Figure 4A). Eight AhAOS genes were with or without one intron (Figure 4B). In addition to AhAOC8, the other eight AhAOC genes contained one or two introns (Figure 4C). All OPR genes had fewer than five introns (Figure 4D). The above results implied that the genes within the same family had similar genetic organization. The high number of introns in the AhLOX gene indicated that the biological functions of this family might be more complex than those of the other three families.

**Figure 5:**
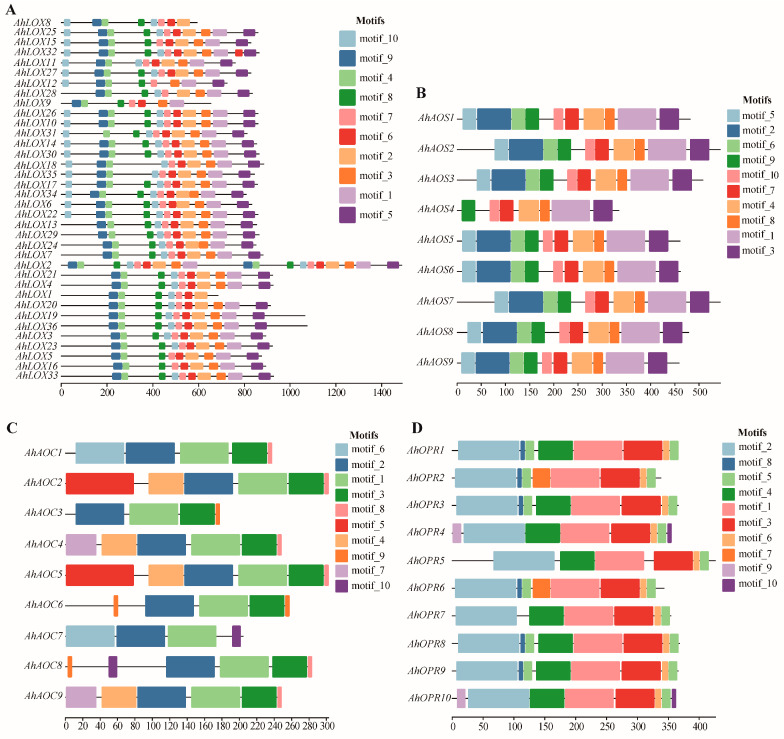
Distribution of conserved motifs of JA biosynthesis-related enzyme genes in *A. hypogaea*. (**A**) AhLOXs; (**B**) AhAOSs; (**C**) AhAOCs; (**D**) AhOPRs. Various conservative motifs are represented by different colors.

To study the structural features of key enzyme proteins of JA biosynthesis, the MEME (Multiple Em for Motif Elicitation) program was utilized to identify the conserved motifs of AhLOXs, AhAOSs, AhAOCs, and AhOPRs in peanut (Figure 5; Tables S6–S9). Ten conserved motifs were detected in each family and distinguished by different colors. The 26 AhLOX proteins contained all motifs. AhLOX9 and AhLOX12 lacked motif 2; AhLOX1, AhLOX8, and AhLOX9 lacked motif 5; motif 6 was not found in AhLOX12 and AhLOX28; AhLOX12 and AhLOX18 lacked motif 8; and AhLOX6, AhLOX9, and AhLOX16 lost motif 10. In the AhAOS family, the composition of conservative motifs is relatively complete. Only AhAOS4 lost motif 2, 5, and 6. Motif 1 and 2 were detected in all AhAOC members. The MEME analysis indicated that only AhAOC7 lacked motif 3 and 7, AhAOC2 and AhAOC5 contained motif 5, AhAOC6 and AhAOC8 contained motif 9, and motif 1, 2, 3, and 8 might be the core components of the Allene_ox_cyc domain. The same trend appeared in the AhOPR family. Motif 1, 2, 3, 5, and 6 were found in all members; AhOPR2 and AhOPR6 lacked motif 4; and motif 7 was detected only in AhOPR2 and AhOPR6. AhOPR4-5, AhOPR7, and AhOPR10 were missing motif 8, and motif 9 and 10 were not discovered in AhOPR4 and AhOPR10. In general, the conserved motifs of protein members within the same family are similar in composition, with some conserved motifs constituting the core components of the characteristic domain, while others show the biological functional diversity of different members.

**Figure 6:**
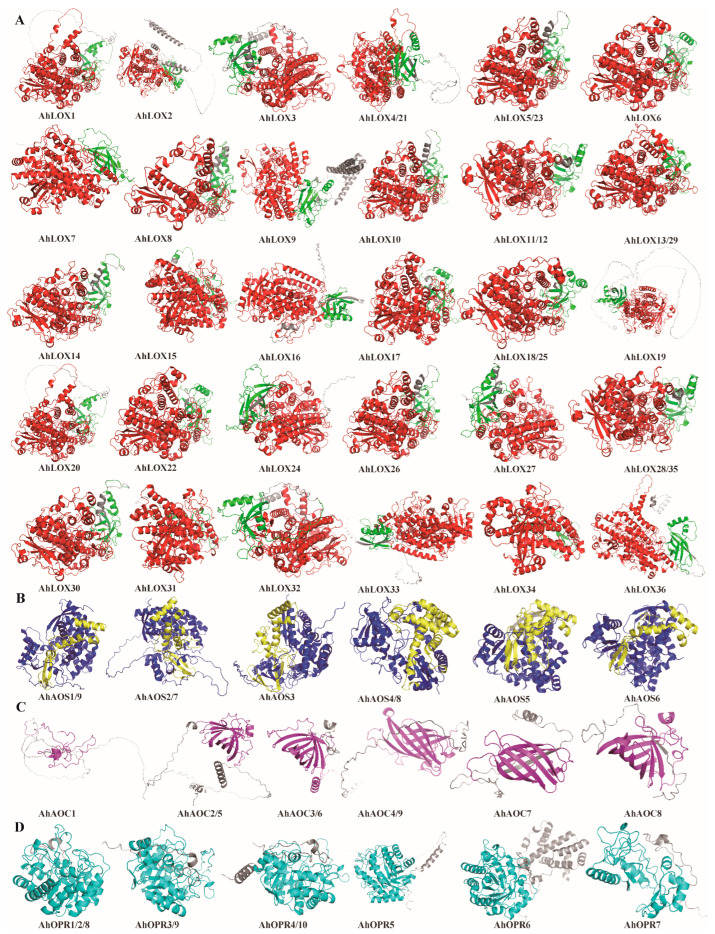
Protein structure and 3D analysis of JA biosynthesis genes in peanut. (**A**) AhLOXs; (**B**) AhAOSs; (**C**) AhAOCs; (**D**) AhOPRs. Three-dimensional structure predictions of 64 proteins; domains are represented by different colors, with PLAT_LH2 in green, lipoxygenase in red, CYP74 in blue, p450 in yellow, Allene_ox_cyc in purple, Oxidored_FMN in light blue, and uncharacterized regions are shown in gray.

Structure and function are compatible, and the structure of a protein determines its biological functional properties. Protein secondary structure prediction showed that all key enzyme proteins of JA biosynthesis have four structures, the alpha helix (Hh), extended strand (Ee), beta turn (Tt), and random coil (Cc), with Cc having the highest percentage of 59.04–36.01% and 46.13–10.04%, followed by Hh and Ee with 10.04–46.13% and 11.65–34.87%, respectively, and Tt with the lowest percentage of 9.76–4.04% (Table S10). In addition, the 3D structures of all proteins were predicted using the protein structure database AlphaFold and visualized using PyMOL software (version 2.5.5; https://pymol.org/2/; accessed on 12 April 2023), and it was found that all proteins contained conserved subunits of subfamilies to which they belonged (Figure 6). The results of the 3D protein structure prediction analyses confirmed that the key enzymes of JA biosynthesis showed a high degree of conservatism among and within the subgroups. The key enzyme proteins of JA biosynthesis were categorized into four types based on their 3D structures and six core structural domains were defined: the PLAT_LH2 structural domain located at the N-terminal and lipoxygenase domain located at the C-terminal of AhLOX protein, the CYP74 structural domain presented in AhAOS protein and p450 domain in the middle, and the Allene_ox_cyc domain at the C-terminal of AOC protein and Oxidored_FMN domain anchored in the middle of OPR protein. This suggested that the higher the amino acid sequence similarity between the proteins, the more similar their 3D structures and the more similar their functions were.

### 2.5. Cis-Regulatory Elements in the Promoter Regions of JA Biosynthesis Genes

**Figure 7:**
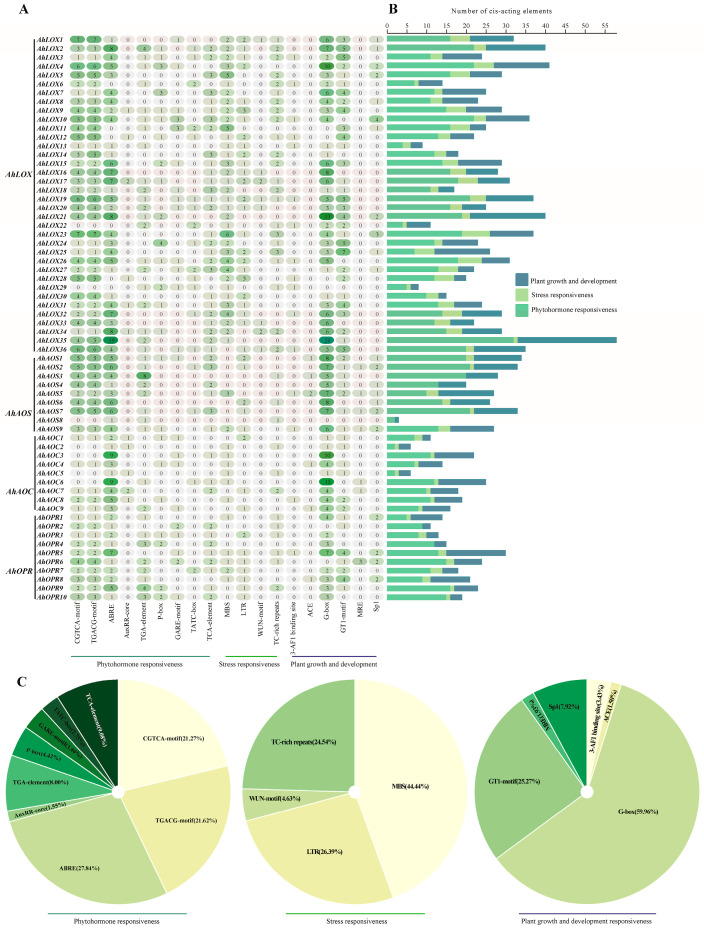
*Cis*-regulatory elements in the promoters of peanut JA biosynthesis-related enzyme genes. (**A**) Distribution of various *cis*-regulatory elements in AhLOX, AhAOS, AhAOC, and AhOPR promoter regions. (**B**) Number of elements in different families is counted. (**C**) Classification and percentage of different *cis*-regulatory elements.

*Cis*-regulatory elements as binding sites of transcription factors connect gene structure and function and play important roles in gene expression [41]. To further analyze the biological function of JA biosynthesis genes, the regions about 2000 bp upstream of the start codon (ATG) were used, analyzing *cis*-regulatory elements of *AhLOX*s, *AhAOS*s, *AhAOC*s, and *AhOPR*s (Figure 7; Table S11). After counting the number of *cis*-regulatory elements, we found that *AhLOX*s had the largest number of elements compared to other families, of which *AhLOX35* contains 57 elements, and 29 *AhLOX* members contain more than 20 elements (Figure 7A,B). The same situation appeared as a result for the *AhAOS* family; 8 *AhAOS* members had more than 20 elements. *AhAOC* members contain a minimum of 6 and a maximum of 25 elements and the number of cis-acting elements in an *AhOPR* member ranges from 11 to 30.

The detected *cis*-regulatory elements could be divided into three categories according to their functional annotations including phytohormone responsiveness, stress responsiveness, and plant growth and development responsiveness (Figure 7C). The CGTCA motif and TGACG motif, as MeJA responsive elements, accounted for 21.27% and 21.62%, respectively. The promoter regions of some genes such as *AhLOX35*, *AhLOX2*, *AhLOX4*, *AhLOX16*, *AhLOX19*, *AhLOX35,* and *AhAOS7* contain a large number of abscisic acid responsive elements, ABRE, AuxRR-core, and TGA-element, suggesting that these genes may be subject to ABA-induced expression. *AhLOX4*, *AhLOX7*, *AhLOX10*, *AhLOX11*, *AhLOX15*, *AhLOX24*, *AhLOX29*, and *AhOPR10* contain more than one gibberellin responsive element including P-box, GARE-motif, and TATC-box. *AhLOX5*, *AhLOX7*, *AhLOX10*, *AhLOX11*, *AhLOX14*, *AhLOX18*, *AhLOX27*, *AhAOS2*, and *AhAOS7* contain three salicylic acid response elements (TCA-element). In total, 44 of the 64 key enzyme genes of JA biosynthesis contained all classes of hormone response elements, and these findings emphasized the possible involvement of these genes in the crosstalk mechanism of various phytohormones.

MBS, a drought responsive element, accounts for 44.44%, which is the largest among all stress-related elements, indicating that these genes may be involved in the response of drought stress signals (Figure 7C). Forty-eight genes contained one or more MBS and forty-three genes contained both ABREs, such as *AhLOX5*, *AhLOX7*, *AhLOX8*, *AhLOX9*, *AhLOX16*, *AhAOS5*, *AhAOC5*, *AhOPR1*, *AhOPR5*, *AhOPR6*, and *AhOPR7*, which signified that these genes may be present in the ABA-dependent drought stress response pathway. Regarding LTR, accounting for 26.39%, 40 genes contained LTRs, of which *AhLOX9*, *AhAOS6*, *AhAOC1*, and *AhOPR3* contained at least two LTRs, demonstrating that JA biosynthesis genes may be induced by a low-temperature signal. *AhLOX1*, *AhLOX17*, *AhLOX19*, *AhLOX3*, and *AhLOX36* had WUN-motif and TC-rich repeats in addition to the above-mentioned, which proved that these genes may take a crucial part in the resistance mechanism of various stresses.

Plant growth and development response elements, including the 3-AF1 binding site, ACE, G-box, GT1-motif, MRE, and Sp1, were also detected in *AhLOX2*, *AhLOX4*, *AhLOX21*, *AhLOX35*, *AhAOS1*, *AhAOS5*, *AhAOC6*, and *AhOPR5*, confirming that these genes may also be involved in peanut growth and development.

### 2.6. Protein–Protein Interactions and miRNA Genes’ Regulatory Networks of JA Biosynthesis Gene Families

**Figure 8:**
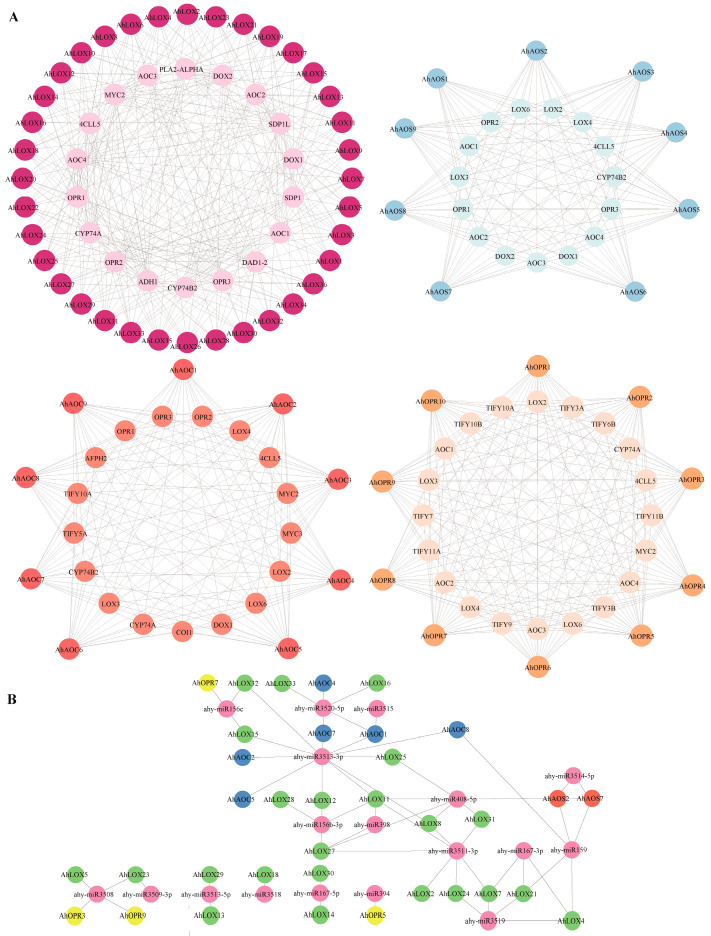
Interactions and regulation networks of genes involved in JA biosynthesis of peanut. (**A**) Predicted protein–protein interaction network of AhLOXs, AhAOSs, AhAOCs, and AhOPRs. (**B**) Regulatory network of putative miRNAs and their targeted candidate genes. Yellow, green, blue, and orange circles represent target genes. The pink circles represent miRNAs. The putative regulatory relationships between miRNAs and their targeted JA biosynthesis-related enzyme genes are shown as gray lines.

To investigate the biological functional properties and metabolic pathways of key enzyme genes of JA biosynthesis and to reveal the possible potential interactions of these proteins, interacting proteins of peanut AhLOXs, AhAOSs, AhAOCs, and AhOPRs were searched and networked by the STRING online database (Figure 8A; Table S12). The analysis revealed that 64 peanut key enzyme proteins of JA biosynthesis interacted with 35 functional proteins. Functional prediction revealed that proteins interacting with peanut key enzyme proteins of JA biosynthesis were mainly involved in the JA biosynthesis pathway, including LOX2-4, LOX6, CYP74A (AOS), hydroperoxide lyase CYP74B2 (HPL1), AOC1-4, and OPR1-3, and transcription factors MYC2, MYC3, jasmonate ZIM-domain protein TIFY3A/JAZ11, TIFY3B/JAZ12, TIFY5A/JAZ8, TIFY5B/JAZ7, TIFY6B/JAZ3, TIFY7/JAZ9, TIFY9/JAZ10, TIFY10A/JAZ1, TIFY10B/JAZ2, TIFY11A/JAZ5, and TIFY11B/JAZ6. Coronatine insensitive 1 (COI1) is mainly involved in hormone response, plant development, and signaling in JA. In addition, peanut key enzyme proteins of JA biosynthesis interacted with 4-coumarate/CoA ligase 5 (4CL5), phospholipase A2-alpha (PLA2-ALPHA), alcohol dehydrogenase 1 (ADH1), defective anther dehiscence 1-2 (DAD1-2), sugar-dependent 1 (SDP1), SDP1-like (SDPL), alpha-dioxygenase 1 (DOX1), and DOX2. These proteins may be involved in lipid synthesis, transport, and catabolism during plant development and reproduction, such as in the hydrolysis of phosphatidylcholine, glycolipids, and triacylglycerols, and may be involved in catalyzing acetaldehyde reduction. Notably, proteins interacting with peanut key enzyme proteins of JA biosynthesis are also involved in responding to external stimuli, stresses, and other phytohormone induction. AhLOX interacts with phospholipase A2-alpha (PLA2-ALPHA) and responds to hormone induction and other external stimuli. All three families (except AhOPRs) interact with DOX1, suggesting that they are involved in defense against oxidative stress due to abiotic stress, prevent oxidative stress and cell death, and can be induced by salicylic acid. AhAOC protein interacted with ABI five binding protein homolog 2 (AFPH2) to mediate germination and response to osmotic stress and sugar stress. The AhOPR protein interacted with nine JAZ proteins, which act as direct targets of SCF/COI1 E3 ubiquitin-ligase and mediate the degradation of this proteasome induced by JA treatment.

To explore the micro-RNA (miRNA) regulation by the JA biosynthesis pathway, the relationship between miRNA-targeted key enzyme proteins of JA biosynthesis and miRNAs was predicted by psRNATarget Server (Figure 8B; Table S13). A total of 16 miRNAs targeted 35 genes, of which 6 LOX and 5 AOC were targeted by 1 miRNA at the same time; ahy-miR3509-3p, ahy-miR3515, ahy-miR3518, and ahy-miR394 could only target 1 gene; and ahy-miR159, ahy-miR3511-3p, ahy-miR3513-3p, ahy-miR3520-5p, and ahy-miR408-5p targeted more than 5 genes, suggesting that these miRNAs may play a key role in the JA biosynthesis pathway.

### 2.7. Expression Profiles of JA Biosynthesis Genes in Different Tissues

**Figure 9:**
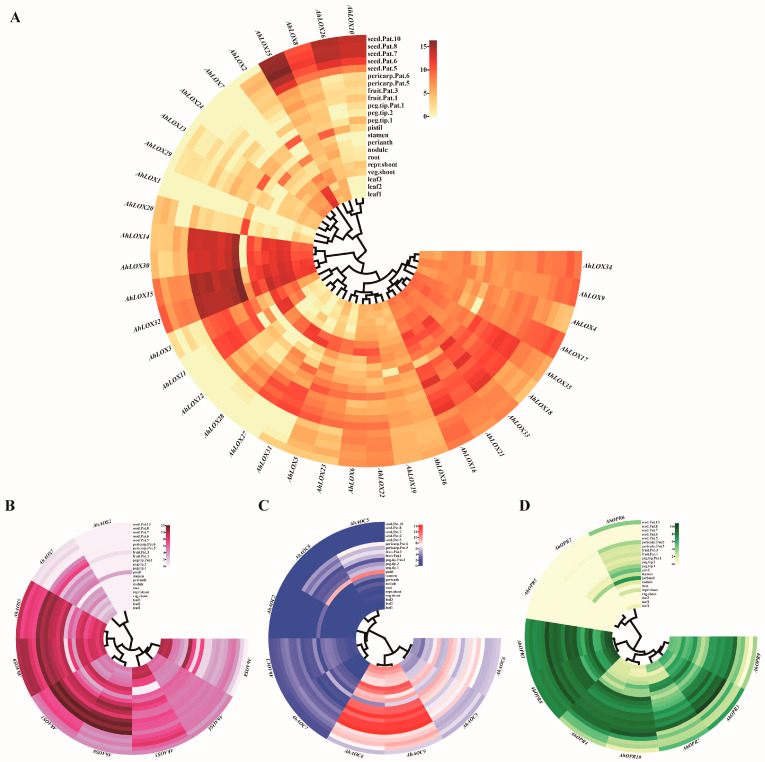
Heatmap of JA biosynthesis-related enzyme genes in 22 peanut tissues by RNA-seq. (**A**) AhLOXs; (**B**) AhAOSs; (**C**) AhAOCs; (**D**) AhOPRs. Leaf 1: Lateral stem leaf; leaf 2: Mainstem leaf; leaf 3: Seedling leaf; veg shoot: Vegetative shoot tip; repr shoot: Reproductive shoot tip; root: Root; nodule: Module; perianth: Perianth; stamen: Stamen; pistil: Pistils; peg tip 1: Peg tip aerial; peg tip 2: Peg tip below soil; peg tip Pat. 1: Peg tip to fruit Pattee 1; fruit Pat. 1: Fruit Pattee 1; fruit Pat. 3: Fruit Pattee 3; pericarp Pat. 5: Pericarp Pattee 5; pericarp Pat. 6: Pericarp Pattee 6; seed Pat. 5: Seed Pattee 5; seed Pat. 6: Seed Pattee 6; seed Pat. 7: Seed Pattee 7; seed Pat. 8: Seed Pattee 8; seed Pat 10: Seed Pattee 10. The relative expression values are shown on left side of heatmap.

To predict the possible biological functions and effects of JA biosynthesis genes on peanut growth and development, RNA-seq data of 22 peanut tissues [42] were utilized, detecting the expression pattern and drawing a heatmap. The analysis verified that most of the genes were expressed to different degrees in 22 tissues (Figure 9; Table S14). *AhAOS2*, *AhAOC2*, *AhOPR2*, and *AhOPR5* were the lower expressed or undetected in most tissues. The heatmap results confirmed that 41 out of 64 peanut JA biosynthesis genes were highly expressed in the leaves, vegetative shoot tip, reproductive shoot tip, and root. Some genes showed tissue specificity; for example, *AhLOX11* was highly expressed in the vegetative shoot tip, reproductive shoot tip, and root; *AhLOX12*, *AhLOX23*, *AhLOX27*, *AhLOX28*, *AhLOX31*, *AhAOC7*, and *AhOPR10* had tissue-specific expression in roots, and these genes were likely to be closely related to stress response.

During peanut growth and development, roots, shoots, leaves, and nodules act as the nutrient organs of the peanut, controlling the transportation of water and nutrients. Among them, roots, shoots, and leaves, as key parts of water transport, are more sensitive to abiotic stress signals. *AhLOX2*, *AhLOX15*, *AhLOX30*, *AhLOX32*, and *AhAOS8* were highly expressed in leaves; *AhLOX14-15*, *AhLOX30*, and *AhLOX32* were highly expressed in shoot tissues. *AhLOX11*, *AhLOX30*, *AhLOX32*, *AhAOC4*, and *AhAOC9* were expressed in the peanut root. *AhLOX11*, *AhLOX14-15*, *AhLOX31*, and *AhLOX32* were expressed in the nodule. This suggested that the above genes played a positive role in the growth and development of peanut and may be involved in the response mechanism to abiotic stresses.

While perianths, stamens, pistils, pegs, pods, and seeds play important reproductive development roles as reproductive organs, *AhLOX3-6*, *AhLOX8-11*, *AhLOX14-19*, *AhLOX21-23*, *AhLOX25-26*, *AhLOX30*, *AhLOX32-36*, *AhAOS1*, *AhAOS3-6*, *AhAOS9*, *AhAOC3-4*, *AhAOC6*, *AhAOC9*, *AhOPR1*, *AhOPR3-4*, and *AhOPR7-10* were highly expressed in these tissues, demonstrating that these genes may control reproductive development of peanut.

### 2.8. Expression Patterns of JA Biosynthesis Genes in Response to Different Abiotic Stress Treatments

**Figure 10:**
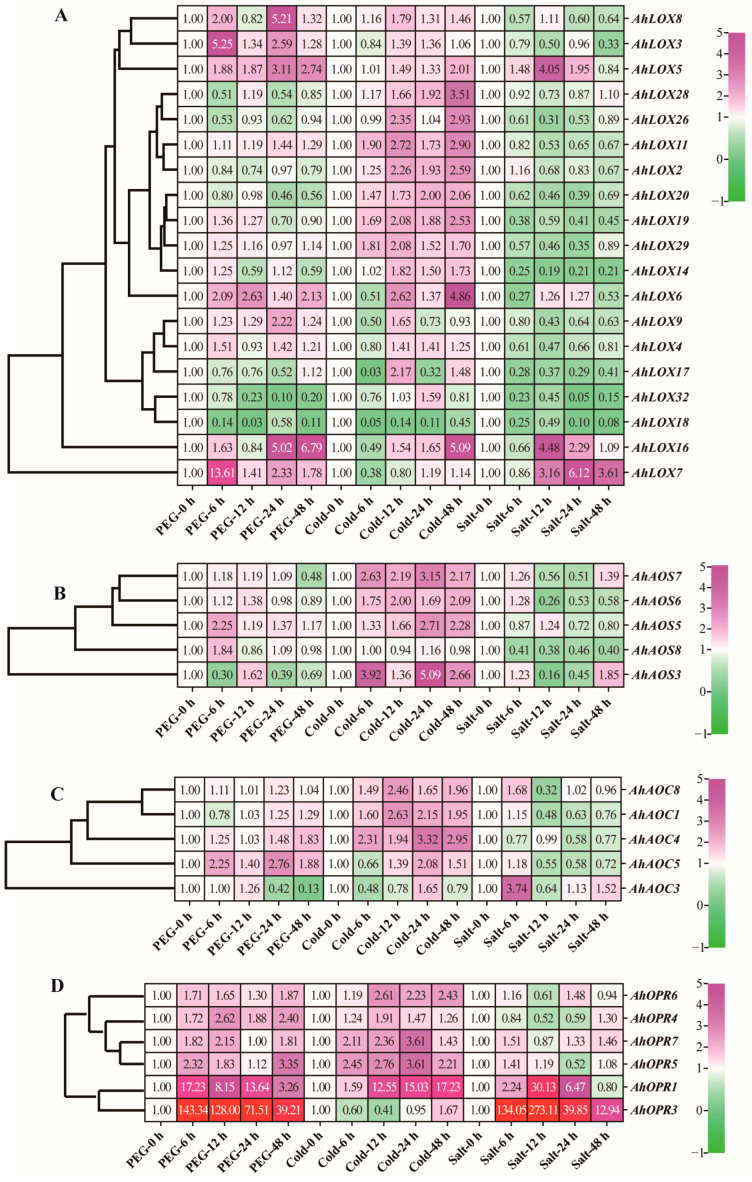
Heatmap of JA biosynthesis-related enzyme genes under drought stress by qRT-PCR. (**A**) AhLOXs; (**B**) AhAOSs; (**C**) AhAOCs; (**D**) AhOPRs. Relative expression values of green (low) to purple (high) are shown in right side of heatmap. Expression levels above 30 times were shown in red.

To investigate whether the expression of JA biosynthesis genes was influenced by abiotic stress and related signaling at the transcriptional level, further expression profiling was discovered under drought, cold, and salt stress for 0 h, 6 h, 12 h, 24 h, and 48 h (Figure 10; Table S15). The results are consistent with our hypothesis; the expression of many JA genes demonstrated significant changes under abiotic stress.

It was found that the expression of most of the genes increased under drought and cold stress treatments. *AhLOX3*, *AhLOX5*, *AhLOX7*, *AhLOX8*, *AhAOS5*, *AhAOS8*, *AhAOC5*, and tested *AhOPR1*, *AhOPR3*, *AhOPR4*, *AhOPR5*, *AhOPR6*, and *AhOPR7* were rapidly up-regulated about 2-fold in expression when they were subjected to drought stress treatment for 6 h, of which *AhOPR1* and *AhOPR3* were up-regulated more than 10-fold at 6 h and *AhOPR3* was even up-regulated more than 100-fold at 6 h, and this trend did not decrease until 48 h. The expression of some genes such as *AhLOX9* and *AhLOX16* did not increase significantly until 24 h after treatment. 

Most of the genes were up-regulated under cold stress. *AhLOX11*, *AhLOX19*, *AhLOX20*, *AhLOX29*, *AhAOS3*, *AhAOS6*, *AhAOS7*, *AhAOC1*, *AhAOC*4, *AhAOC8*, *AhOPR5*, and *AhOPR7* were up-regulated about 2-fold at 6 h under cold stress; *AhLOX2*, *AhLOX6*, *AhLOX11*, *AhLOX17*, *AhLOX19*, *AhLOX26*, *AhLOX29*, *AhAOS7*, *AhAOC1*, *AhAOC8*, *AhOPR1*, and *AhOPR5-7* were up-regulated to more than 2-fold transcript levels at 12 h of cold stress treatment compared to the control. The expression of *AhLOX20*, *AhAOS5*, *AhAOS7*, *AhOPR1*, *AhAOC1*, *AhAOC4-5*, *AhAOS3*, and *AhOPR5-7* was up-regulated more than 2-fold at 24 h of cold stress treatment, with more than a 5-fold up-regulation of *AhOPR1* and *AhAOS3*. The transcript levels of *AhLOX2*, *AhLOX5-6*, *AhLOX11*, *AhLOX16*, *AhLOX19-20*, *AhLOX26*, *AhLOX28*, *AhAOS3*, *AhAOS5-7*, *AhAOC4*, *AhOPR1*, and *AhOPR5-6* were more than 2-fold compared to those of the control at 48 h of cold stress, with more than 5-fold for *AhOPR1* and *AhLOX16*. It is a remarkable fact that the *AhOPR1*, which performed well under drought stress, showed a trend of up-regulation at 12 h, while *AhOPR3* was not slightly expressed until 48 h. 

*AhLOX7* and *AhLOX16* were down-regulated at 6 h, followed by an overall up-regulated trend. *AhLOX5*, *AhLOX6*, *AhLOX7*, *AhAOS3*, *AhAOC3*, *AhAOC8*, *AhOPR1*, and *AhOPR3* transcript levels were up-regulated under salt stress treatments, with *AhAOC3*, *AhAOC8*, *AhOPR1*, and *AhOPR3* rapidly up-regulated at 6 h, while the remaining genes were not significantly up-regulated (less than 1.5-fold) or down-regulated. Notably, *AhOPR1* and *AhOPR3* were transcribed at more than a 30-fold level at 12 h of salt stress. *AhLOX8*, *AhLOX18-19*, *AhLOX32*, *AhAOS5*, and *AhAOC4* were down-regulated at 6 h under salt stress, up-regulated after 12 h, and down-regulated again at 24–48 h. *AhLOX17*, *AhLOX20*, *AhLOX26*, *AhLOX29*, and *AhOPR4* were rapidly downgraded at 6 h and not upgraded to around background levels until 48 h. These results suggested that these genes may be negative regulators in the pathway against salt stress and that some of the genes may be upstream and can cascade signals.

Taken together, the results indicated that most of the genes were up-regulated in transcript levels under drought, cold, and salt stresses, which might be involved in the defense mechanism against stress signals, and some genes were down-regulated in expression, which might play the role of negative feedback regulation. Individual genes were first up-regulated and then down-regulated, which may be involved in the transduction and regulation of stress signals. Interestingly, these key enzyme genes of JA biosynthesis (except *AhOPR1* and *AhOPR3*) were expressed completely differently under different abiotic stress treatments, and did not appear to be highly expressed under both stresses at the same time, suggesting that these genes may exercise different biological functions in response to different abiotic stresses.

## 3. Discussion

Plant growth, development, and survival depend on the natural environment in a complex and strictly interconnected network, and environmental factors unfavorable to growth and survival become the main threats to this network [43]. Among these environmental factors, abiotic stresses represented by drought, extreme temperatures, and saline conditions are considered as the main abiotic sources of stimuli [44]. Plants have developed complex mechanisms to resist and mitigate unfavorable environments over a long evolutionary process, including the intrinsic regulation of phytohormones [45]. JA, as an important signaling and regulatory molecule, affects plant growth, development, and yield, including the regulation of stomatal opening and closing, cell cycle, and uptake of glucose and nutrients such as nitrogen and phosphorus [2,8]. Meanwhile, JA is involved in the perception and transmission of various environmental signals and makes defense responses to adversity stress, in which the key enzyme genes of the JA biosynthesis pathway play a crucial leading role [46]. Therefore, a genome-wide analysis of gene families of key enzymes for JA biosynthesis can be an effective tool for mining key resistance genes and exploring mechanisms of resistance to abiotic stresses.

The genome-wide identification and bioinformatics analysis of LOX, AOS, AOC, and OPR have been studied in different species such as *Arabidopsis*, rice, wheat, maize, and soybean [47,48,49,50,51,52,53]. In this study, key enzyme genes of JA biosynthesis within the genomes of two wild species of peanut, cultivated peanut, model plants of *Arabidopsis thaliana*, rice, maize, and near-origin species of soybean, chickpea, and common bean were rescreened and identified by a hidden Markov model search, and all members were re-determined based on the results of existing studies and structural domain searches [28,54,55,56]. Although the cultivated peanut is a heterotetraploid cross between two wild species of peanut, the number of key enzyme genes of JA biosynthesis did not follow this principle exactly; not all AOS, AOC, and OPR genes on the allotetraploid peanut genome find their corresponding homolog genes in the A and B genome, and the total number of them is less than the sum of the number of genes within the A and B genomes. This may be due to the fact that these gene families lost some genes during the evolutionary process, or some of the functionally redundant genes were discarded while a doubling replication event occurred. A similar situation was found in other gene families of peanut. The natural resistance-associated macrophage protein (NRAMP) family has 7 *AdNRAMP*s, 6 *AiNRAMP*s, and 15 *AhNRAMP*s (Tan et al., 2023). In addition, only 14 *AdSAUR*s and 19 *AiSAUR*s were found, the number of which was much smaller than that of *AhSAUR*s (162) [57]; 68, 36, and 31 major latex-like proteins (MLPs) were obtained from *A. hypogaea*, *A. duranensis*, and *A. ipaensis*, respectively [58]. 

However, studies on key enzyme genes of JA biosynthesis within a single species are limited. In this study, we conducted the first comprehensive and systematic identification and bioinformatics analysis of peanut key enzyme genes of JA biosynthesis, and explored their roles in the stress resistance mechanism of peanut through the application of abiotic stresses (drought stress, cold stress, and salt stress). A phylogenetic relationship analysis with key enzyme genes of JA biosynthesis in the genomes of other species revealed that members of the four families, LOX, AOS, AOC, and OPR, in peanut were all more related to soybean, chickpea, and common bean key enzyme genes of JA biosynthesis, which may be attributed to the fact that peanut belongs to the same family of legume plants as these three species. Overall, members of each family contain conserved structural domains characteristic of the family, notably the presence of two lipoxygenase domains in AhLOX2 (Figure 3). 

Initially, we hypothesized that one additional conserved domain might enhance its defense against abiotic stresses or be able to respond faster than other members, but subsequent qRT-PCR results showed that it did not perform well (Figure 8). Differences in the number and mode of insertion of introns in the gene structure, as well as the type and number of conserved motifs, may lead to genes with different biological functions. In our study, the number of introns in AhLOX family members is around 6–14, while the number of introns in AhAOS, AhAOC, and AhOPR family members is less than 5, which indirectly proves the complexity and diversity of functions of AhLOX family members. Through the conserved motif analysis, we found that 26 out of 36 members of the AhLOX family have similar conserved motif composition, and the conserved motif composition of the AhAOS, AhAOC, and AhOPR families is relatively complete, with only some of the members missing 1–2 motifs, which confirms that the same family has similar biological functions, but the difference of some motifs leads to the convergence of the functions.

Based on the results of a bioinformatic analysis, including a *cis*-regulatory element analysis, protein–protein interaction analysis, and tissue expression analysis, 35 potential responsive abiotic stress genes were screened and their expression under drought, cold, and salt stresses was examined by qRT-PCR. A large number of *cis*-regulatory elements responsive to abiotic stresses are present in the promoter regions of these genes, which are highly expressed in water-transporting tissues and are predicted to interact with proteins that defend against abiotic stresses. Inconsistent with our expectation, it was found that most of the genes were not up-regulated under these three stress conditions as we expected. In yellow horn, *XsLOX1-2*, *XsLOX5-8*, and *XsLOX10* were up-regulated in cold stress and down-regulated after salt stress treatment [59]. In wheat, *TaLOX16* and *TaLOX18* were up-regulated after drought stress and down-regulated under salt stress [53]. In this study, *AhLOX17*, as a homolog gene of *TaLOX16* and *TaLOX18*, was first down-regulated and then slightly up-regulated under drought stress treatment and down-regulated under salt stress treatment. A total of 10 *GhOPR* genes were discovered in cotton, which showed opposite expression trends under drought stress and salt stress, among which *GhOPR1-2*, *GhOPR4-5*, *GhOPR8*, and *GhOPR9* were down-regulated under drought stress, and then up-regulated and then down-regulated under salt stress. *GhOPR3*, *GhOPR6*, and *GhOPR10* were down-regulated under drought stress and remained up-regulated under salt stress. *GhOPR7* was up-regulated under drought stress and then down-regulated under salt stress [60]. It can be hypothesized from these results that key enzyme genes of JA biosynthesis respond differently in the face of different abiotic stresses, and that some genes that exhibit resistance under one stress may turn into negative regulators involved in stress signaling when under another stress, thereby engaging more functional genes in resistance and preventing functional redundancy.

**Figure 11:**
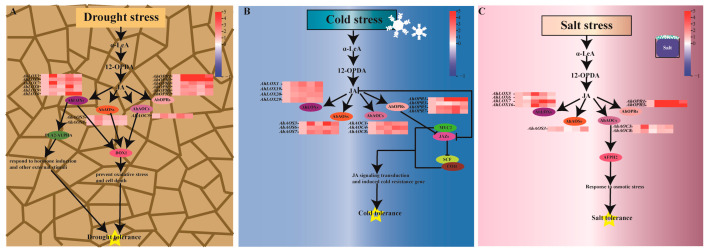
Key enzyme proteins of JA biosynthesis-mediated drought (**A**), cold (**B**), and salt stress (**C**) responses in peanut. Solid arrows represent direct promotion and solid horizontal bars represent suppression of expression. Gene expression range increasing from blue to red and placed on right side of figure. Solid arrows indicate promotion.

To deepen the understanding of the JA biosynthesis pathway, this study presents a schematic diagram of the JA biosynthesis pathway to gain insights into the key genes involved in defense against abiotic stresses and their potential roles (Figure 11). Under drought stress, *AhLOX3*, *AhLOX5*, *AhLOX7*, *AhLOX8*, *AhLOX9*, and *AhLOX16* in the JA biosynthesis pathway were induced to be up-regulated and expressed, together with PLA2-ALPHA interacting with them, in response to phytohormone induction and other exogenous stimuli. The expression of *AhAOS5*, *AhAOS8*, and *AhAOC4* was also rapidly elevated, and they interacted with DOX1, thereby protecting against oxidative stress and accompanying cell death (Figure 11A). When cold stress occurred, JA biosynthesis was inhibited; *AhOPR1*, *AhOPR3*, *AhOPR5*, and *AhOPR7* were induced to be up-regulated for expression along with *AhLOX*s, *AhAOS*s, and *AhAOC*s; and increased JA concentration may have inhibited the *AhJAZ*s that interact with them, releasing *AhMYC2* to initiate the transport JA signaling and induce cold resistance genes (Figure 11B). *AhAOC*s are critical in the mechanism of resistance to salt stress. Upon exposure to salt stress, *AhAOC3* and *AhAOC8* transcript levels were up-regulated and interacted with *AFPH2* in response to osmotic stress (Figure 11C).

## 4. Materials and Methods

### 4.1. Identification of Key Enzyme Genes of JA Biosynthesis in Peanut

Peanut genome and annotation files were obtained from a peanut genomics resource (https://peanutbase.org/; accessed on 26 June 2023). The key enzyme gene family of JA biosynthesis in the *Arachis hypogaea* genome was identified by the HMMER-3.0 program. The hidden Markov model (HMM) profiles of the lipoxygenase (PF00305) and PLAT_LH2 domain (PF01477) of LOXs, P450 domain (PF00067) of AOSs, Allene_ox_cyc domain (PF06351) of AOCs, and Oxidored_FMN domain (PF00724) of OPRs were retrieved from the Pfam website and were used to identify the putative proteins with the best domain e-value cutoffs of <1 × 10^−3^. To validate the accuracy of these predicted genes, we utilized the online tool SMART (http://smart.embl-heidelberg.de/; accessed on 6 January 2024), pfam (version 36.0; http://pfam.xfam.org/; accessed on 3 January 2024), and NCBI-CDD (https://www.ncbi.nlm.nih.gov/cdd; accessed on 6 January 2024) to confirm that all candidate genes had conserved domains [61,62]. The online tool ProtParam (http://web.expasy.org/protparam/; accessed on 7 January 2024) was utilized, analyzing typical characters, including the theoretical isoelectric point (PI), the instability index, the aliphatic index, and the molecular weight. The Plant-mPLoc online database (http://www.csbio.sjtu.edu.cn/bioinf/plant-multi/; accessed on 15 January 2024) was utilized, predicting the subcellular localization of LOX, AOS, AOC, and OPR proteins [63]. The amino acid, genome, and coding sequences of key enzyme genes of JA biosynthesis in *A. duranensis*, *A. ipaensis*, and *A. hypogaea* are shown in Table S16.

### 4.2. Gene Structure and Conserved Motifs and Protein Structures

The Gene Structure Display Server (GSDS 2.0) (http://gsds.gao-lab.org; accessed on 10 December 2023) was used, analyzing the composition of exons and introns based on the peanut genome annotation file and to display the structure [64]. MEME [65] was applied to predict the conserved motifs with the following parameter settings: number of repetitions—any, maximum number of motifs—10, minimum motif width—6, and maximum motif width—100. Graphic beautification was performed by TBtools software (version v2.096; https://github.com/CJ-Chen/TBtools-II/releases; accessed on 12 May 2024) [66]. Protein secondary structure prediction was performed by the online tool SOPMA (https://npsa-pbil.ibcp.fr/cgi-bin/npsa_automat.pl?page=npsa_sopma.html; accessed on 6 March 2024), and the 3D structures of all key enzyme proteins of JA biosynthesis were predicted using the protein structure database AlphaFold (https://alphafold.ebi.ac.uk/; accessed on 26 October 2023) and visualized using PyMOL software (version 2.5.5; https://pymol.org/2/; accessed on 12 April 2023).

### 4.3. Phylogenetic Analysis and Classification

To understand the phylogenetic relationship and to classify the key enzyme genes of JA biosynthesis in peanut, maximum-likelihood trees were constructed based on the full-length amino acid sequences of LOXs, AOSs, AOCs, and OPRs from *Arachis duranensis*, *Arachis ipaensis*, *Arachis hypogaea*, *Arabidopsis thaliana*, *Oryza sativa*, *Zea mays*, *Glycine max*, *Cicer arietinum*, *and Phaseolus vulgaris* (https://phytozome-next.jgi.doe.gov/; accessed on 16 April 2024) [67]. All of the above proteins have been identified to contain the characteristic structural domains of the family by the HMMER web server (https://www.ebi.ac.uk/Tools/hmmer/; accessed on 28 April 2024) [38]. Phylogenetic trees were constructed by MEGA11 software [68].

### 4.4. Chromosomal Location

Key enzyme genes of JA biosynthesis were mapped to the chromosomes of *Arachis duranensis*, *Arachis ipaensis*, and *Arachis hypogaea*, according to genome annotation GFF files (https://phytozome-next.jgi.doe.gov/; accessed on 16 April 2024), and the results were visualized by MG2C online software (http://mg2c.iask.in/mg2c_v2.1/; accessed on 16 March 2024) [69].

### 4.5. Cis-Regulatory Element Analysis of Promoter Region

*Cis*-regulatory elements as binding sites of transcription factors connect gene structure and function and play important roles in gene expression. The *cis*-regulatory elements in key enzyme gene promoter regions of JA biosynthesis were predicted using PlantCARE (http://bioinformatics.psb.ugent.be/webtools/plantcare/html/; accessed on 19 April 2024) [70] and visualized by TBtools software (version v2.096; https://github.com/CJ-Chen/TBtools-II/releases; accessed on 12 May 2024). The promoter sequences of key enzyme genes of JA biosynthesis in *A. hypogaea* are shown in Table S17.

### 4.6. Protein–Protein Interaction and miRNA Genes’ Regulatory Network

The interaction of key enzyme proteins of JA biosynthesis with Arabidopsis proteins was predicted by the online database STRING (https://version-12-0.string-db.org/; accessed on 18 April 2024) with a confidence threshold set at 0.4 and visualized by native software Cytoscape 3.7.2 (version 3.7.2). Peanut miRNA sequences were obtained from the online database miRbase (http://www.mirbase.org/; accessed on 18 April 2024). Extracted cDNA sequences were used, predicting miRNA targets, and miRNA regulatory network maps were constructed using Cytoscape software (version 3.7.2) [71].

### 4.7. Tissue Expression Pattern

The expression of JA genes in 22 tissues in allotetraploid peanut was obtained from the peanut genome resource PeanutBase (https://peanutbase.org/; accessed on 18 January 2024), FPKM values were calculated and normalized, and genes with the same expression pattern within each family were clustered and visualized using TBTools software (version v2.096; https://github.com/CJ-Chen/TBtools-II/releases; accessed on 12 May 2024).

### 4.8. Plant Materials and Abiotic Stress Treatment

Mature seeds of *A. hypogaea* L. Cultivar NongHua 5 (NH5) were provided by the Peanut Research Institute of Shenyang Agricultural University. Peanut seeds of uniform size, fullness, and viability were selected, sterilized by 75% ethanol (*v*:*v*) for 10 s, and soaked in deionized water for 12 h. The seeds were sown into individual pots (10 cm in diameter and 20 cm in depth) containing vermiculite and nutrient soil (1:1; *v*:*v*), and placed in an intelligent artificial climate incubator for cultivation at the following conditions: temperature of 28 °C/23 °C (day/night), light duration of 16 h/8 h (day/night), light intensity of 600 μmol m^−2^ s^−1^, and 60% relative humidity. When the seedlings were cultivated to the three-leaf–one-center stage, seedlings with consistent growth were selected for abiotic stress treatment. Drought stress: seedlings were removed from the substrate, cleaned with deionized water and dried, and immersed in a 20% (*v*:*v*) PEG-6000 solution. Salt stress: seedlings were incubated in a 200 mmol·L^−1^ sodium chloride (NaCl) solution. Cold stress: the incubator was set at 6 °C/6 °C (day/night). Samples were collected from the third leaves from top to bottom at 6 h, 12 h, 24 h, and 48 h, respectively, with 0 h as the control. Three biological and three technical replicates were set up. Seedlings were quickly frozen in liquid nitrogen and stored at −80 °C.

### 4.9. RNA Extraction and Gene Expression Analysis under Abiotic Stress

RNA was extracted by the Trizol–chloroform method, and all RNAs were analyzed by agarose gel electrophoresis, and then the RNA concentration was determined by a Nanodrop ND-1000 spectrophotometer. The reverse transcription PCR system was as follows: 5×RT Buffer at 10 μL, RT Enzyme Mix at 2.5 μL, dNTPs at 2.5 μL, Oligo (dT) at 1.25 μL, random hexamers at 1.25 μL, and Template RNA at 3 μg; and the system was supplemented with RNase-free ddH_2_O to 50 μL. The reaction was carried out as follows: 50 °C for 60 min and 85 °C for 5 min. Primers were designed for the coding sequences of 35 genes of the key enzyme gene family of JA biosynthesis with Ahactin11 as an internal reference, and the length of primers was limited to 80–200 bp. qRT-PCR amplification was performed in the following system: 10 μL of template cDNA, 0.1 μL of each primer in the forward and reverse directions, 5 μL of 2×SYBR Green qPCR Master Mix, and 4.8 μL of ddH_2_O. The reaction was carried out at 95 °C for 10 min, followed by 40 cycles at 95 °C for 15 s and 60 °C for 30 s. Processing of qRT-PCR data was performed by the 2^−△△CT^ method. Primer sequences are shown in Table S18.

## 5. Conclusions

In conclusion, 64 key enzyme genes of JA biosynthesis were identified and classified according to phylogenetic relationship, gene structure, conserved motif, and protein structure analyses in peanut. They had a large number of growth and development-associated, abiotic stress responsive, and phytohormone responsive *cis*-regulatory elements and interacted with proteins that defend against abiotic stresses. Five miRNAs were found to target more than five genes, suggesting that these miRNAs may play a key role in the JA biosynthesis pathway. *AhLOX32* was highly expressed in perianths, stamens, pistils, pegs, pods, and seeds that affect the reproductive development of peanut. *AhLOX16*, *AhOPR1*, and *AhOPR3* were up-regulated for expression after drought stress treatment. *AhAOS3*, *AhOPR1*, and *AhAOC4* had elevated transcript levels in response to cold stress treatment. *AhLOX5*, *AhLOX16*, *AhAOC3*, *AhOPR1*, and *AhOPR3* were up-regulated for expression under salt stress. The data obtained in this study came from the artificial climate incubator, and the results need to be verified by field experiments. The results of these analyses provide a new vision to study the regulation of JA biosynthesis in peanut against abiotic stress, and the candidate genes screened based on a bioinformatics analysis also provide a theoretical basis for peanut to resist adversity.

## Data Availability

All data in this study are available in this article and its Supplementary Files.

## Supplementary Materials

The following supporting information can be downloaded at: https://www.mdpi.com/article/10.3390/ijms25137054/s1, Table S1: Basic information on LOX family members in *A. duranensis*, *A. ipaensis* and *A. hypogaea*; Table S2: Basic information on AOS family members in *A. duranensis*, *A. ipaensis* and *A. hypogaea*; Table S3: Basic information on AOC family members in *A. duranensis*, *A. ipaensis* and *A. hypogaea*; Table S4: Basic information on OPR family members in *A. duranensis*, *A. ipaensis* and *A. hypogaea*; Table S5: Sequence similarity of JA biosynthesis homologous gene pair in *A. duranensis*, *A. ipaensis* and *A. hypogaea*; Table S6: The sequences of the 10 motifs by motif analysis of AhLOXs; Table S7: The sequences of the 10 motifs by motif analysis of AhAOSs; Table S8: The sequences of the 10 motifs by motif analysis of AhAOCs; Table S9: The sequences of the 10 motifs by motif analysis of AhOPRs; Table S10: Proportion of protein secondary structure of AhLOXs, AhAOSs, AhAOCs and AhOPRs; Table S11: Quantity statistics of different cis-acting elements presented in AhLOX, AhAOS, AhAOC and AhOPR promoters; Table S12: Details of the proteins that interacted with AhLOXs, AhAOSs, AhAOCs and AhOPRs in protein-protein interaction network; Table S13: Details of the targeting relationship between AhLOXs, AhAOSs, AhAOCs, AhOPRs and miRNA; Table S14: The expression levels of AhLOXs, AhAOSs, AhAOCs and AhOPRs in 22 different tissues; Table S15: The relative expression levels of AhLOXs, AhAOSs, AhAOCs and AhOPRs under multiple abiotic stresses; Table S16: The amino acid, genome and coding sequences of key enzyme genes of JA biosynthesis in *A. duranensis*, *A. ipaensis* and *A. hypogaea*; Table S17: The promoter sequences of key enzyme genes of JA biosynthesis in *A. hypogaea*; Table S18: Quantitative primers of AhLOXs, AhAOSs, AhAOCs, AhOPRs.
